# Two Cases of Lung Abscess and Pleuritis in Severe COVID-19 Patients

**DOI:** 10.7759/cureus.61614

**Published:** 2024-06-03

**Authors:** Shiho Goda, Tatsuya Yuba, Kohei Yamamoto, Misaki Sasakura, Noriya Hiraoka

**Affiliations:** 1 Department of Respiratory Medicine, Japanese Red Cross Kyoto Daiichi Hospital, Kyoto, JPN; 2 Department of Infection Control, Japanese Red Cross Kyoto Daiichi Hospital, Kyoto, JPN

**Keywords:** pleuritis, lung abscess, secondary bacterial infection, tocilizumab, covid-19

## Abstract

We report two patients who were treated with remdesivir, steroids, and tocilizumab for severe coronavirus disease 2019 (COVID-19) and developed lung abscesses and pleuritis. Although complications due to bacterial infections are often reported in COVID-19 patients, these severe infections are rare. Patients receiving tocilizumab are at a high risk of developing serious bacterial infections, and the diagnosis is often delayed because symptoms such as fever and elevated C-reactive protein levels are often minimal. The possibility of complications owing to severe bacterial infections should be considered when treating patients with severe COVID-19.

## Introduction

In December 2019, an outbreak of pneumonia was reported in Wuhan, Hubei Province, China. Severe acute respiratory syndrome coronavirus 2 (SARS-CoV-2) was identified as the causative virus and was named coronavirus disease 2019 (COVID-19). The first case was reported in Japan in January 2020. Since then, the explosive spread of the infection has continued. Bacterial superinfections are often observed in COVID-19 patients [1,2]. However, while only a few cases of lung abscesses have been reported [3,4], instances of pleurisy and pyothorax have not been documented, and occurrences leading to these severe bacterial infections are rare. In this report, we describe a case of lung abscess and bacterial pleurisy due to severe COVID-19 infection.

The abstract of this article was presented at the 239th Kinki Regional Meeting of the Japanese Society of Internal Medicine held on March 4, 2023.

## Case presentation

Case 1

A 77-year-old, previously healthy, thin (body mass index (BMI): 17.5 kg/m^2^), past smoking man developed a fever. The following day, a SARS-CoV-2 antigen qualitative test was positive. On day five, due to dyspnea, he was transferred to another hospital. His arterial blood oxygen level was 59 Torr with 15 L/minute of oxygen through a reservoir mask. Subsequently, he was intubated, started on a ventilator, transferred to our hospital, and admitted the same day. Upon arrival, he had no fever. Blood laboratory data showed a C-reactive protein (CRP) level of 49.17 mg/dL.

**Figure 1:**
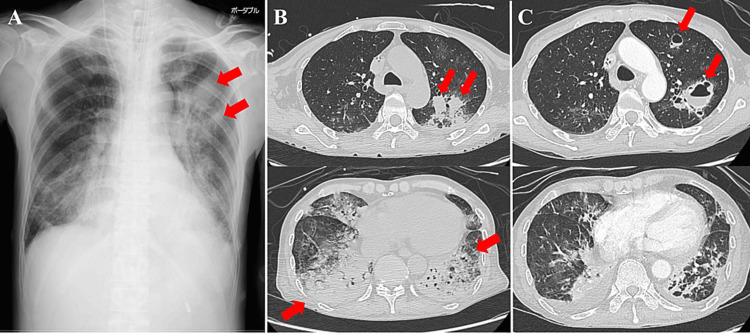
Chest radiography and CT images for case 1. (A) Chest radiography upon admission for the patient in case 1 revealing consolidation in the right lower and entire left lung fields. Two nodules were observed in the left lung fields (red arrows). (B) Chest CT images upon admission for the patient in case 1 revealing extensive consolidation in the right middle and lower lobes and the entire left lobe (red arrows). (C) Contrast-enhanced chest CT images on day 13 for the patient in case 1 revealing several pulmonary abscesses in the left upper lobe (red arrows). Consolidations in the bilateral lower lobes improved.

Chest radiography on day five (Figure 1, Panel A) showed consolidation in the right lower and left whole-lung fields and two nodules in the left lung fields. Chest computed tomography (CT) revealed extensive consolidation in the right middle and lower lobes and the entire left lobe (Figure 1, Panel B). Methicillin-sensitive *Staphylococcus aureus* (MSSA) and *Klebsiella pneumoniae* were detected in the sputum cultures, whereas MSSA was detected in only one set of blood cultures.

He was administered remdesivir, methylprednisolone, tocilizumab, and ceftriaxone (CTRX) on the day of admission. The inflammatory parameters and respiratory condition improved, and he was extubated on day 11. The treatment for COVID-19 ended on day 14.

**Figure 2:**
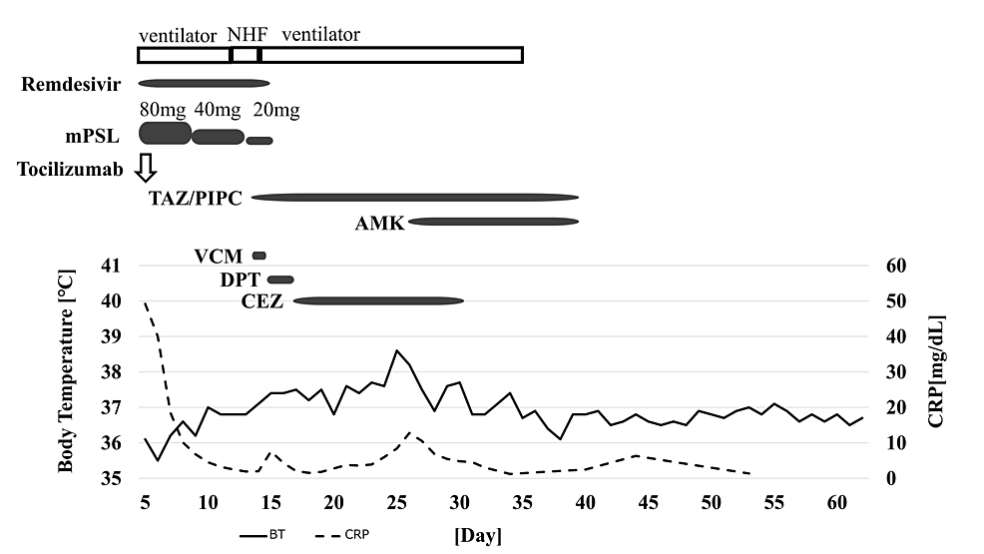
Clinical course for case 1. The day of symptom onset is designated as day 0. NHF: nasal high flow; mPSL: methylprednisolone; TAZ/PIPC: tazobactam/piperacillin; AMK: amikacin; VCM: vancomycin; DPT: daptomycin; CEZ: cefazolin; BT: body temperature; CRP: C-reactive protein

However, chest radiography and CT revealed several pulmonary abscesses in the left upper lobe on day 13 (Figure 1, Panel C). His respiratory condition deteriorated again on day 14, and he was reintubated. On day 14, tazobactam/piperacillin and vancomycin (VCM) were administered empirically. MSSA was detected in the blood culture on day 14 and *Pseudomonas aeruginosa* in the sputum culture on day 16. VCM was changed to daptomycin (DPT) on day 16 because the source of the infection was a suspected catheter infection. Afterward, DPT was de-escalated to cefazolin (CEZ) because the bacteria were found to be MSSA. Amikacin was added on day 25 due to persistent *P. aeruginosa* retention. Antimicrobial therapy was terminated on day 38 as his condition improved. A tracheostomy was performed on day 24. He was weaned from the ventilator because his respiratory status improved as the lung abscesses improved. He was transferred to another hospital on day 62 for rehabilitation. Figure 2 shows his clinical course from admission to discharge.

Case 2

**Figure 3:**
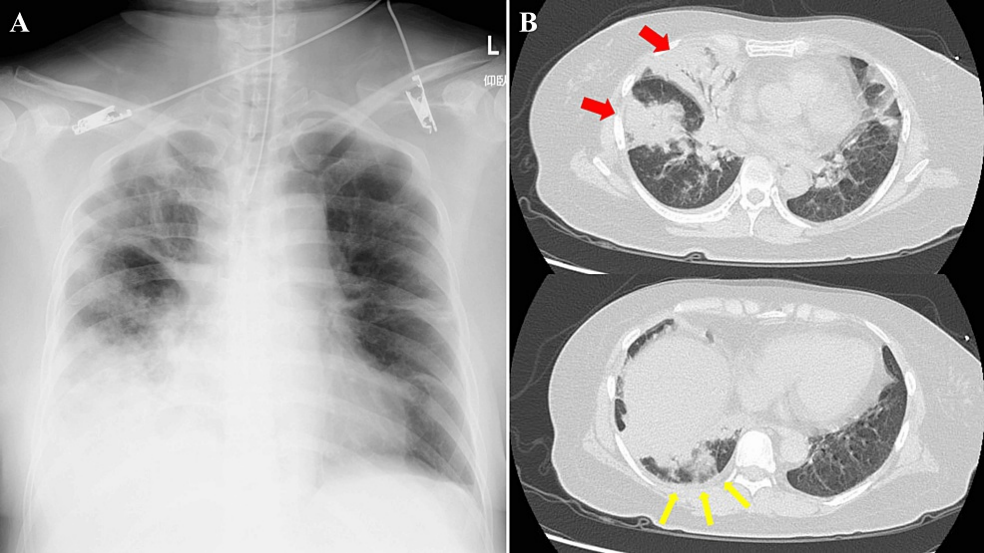
Chest radiography and CT images for case 2. (A) Chest radiography upon admission for the patient in case 2 revealing consolidation in the right upper and lower lung fields. (B) Chest CT image upon admission for the patient in case 2 revealing extensive consolidation throughout the right lobe (red arrows) and very few pleural effusions in the right thoracic cavity (yellow arrows).

A 58-year-old, non-smoking woman with diabetes, hypertension, hyperthyroidism, and obesity presented with a fever. The SARS-CoV-2 antigen qualitative test was found to be positive. On day eight, she was brought to the hospital because of severe right chest pain. She was intubated and started on a ventilator because she could not maintain oxygenation even with a 10 L oxygen mask. Because she could not maintain her blood pressure, she was administered 0.03 μg/kg/minute of noradrenaline and transferred to our hospital. Upon arrival at our hospital, she developed acute type 1 respiratory failure with a P/F ratio of 112. Blood laboratory data showed a CRP level of 39.66 mg/dL. Chest radiography (Figure 3, Panel A) revealed consolidation in the right upper and lower lung fields.

A CT (Figure 3, Panel B) revealed extensive consolidation throughout the right lobe and very few pleural effusions in the right thoracic cavity. She started treatment with remdesivir, dexamethasone, and tocilizumab on day eight and CTRX. Dexamethasone was discontinued on day 11 due to poor glycemic control and improved respiratory status. On day 12, MSSA was detected in sputum culture and two sets of blood cultures on admission. She received CEZ for MSSA bacteremia on day 12. She was extubated on day 18. However, her right pleural effusion significantly increased, and the thoracic cavity appeared multifocal on ultrasound on day 20. The pleural effusion sample was neutrophilic effusion, but no bacteria were detected.

**Figure 4:**
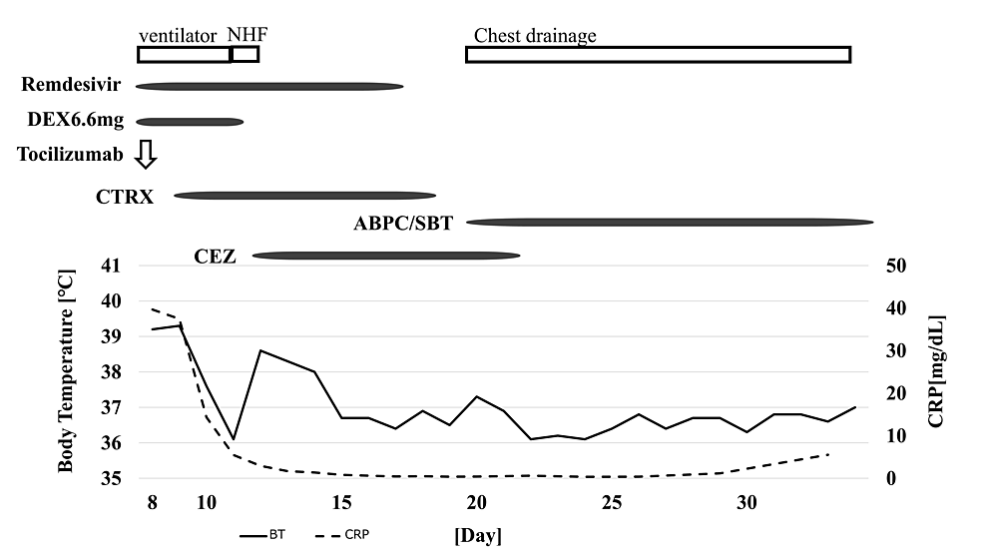
Clinical course for case 2. The day of symptom onset is designated as day 0. NHF: nasal high flow; DEX: dexamethasone; CTRX: ceftriaxone; ABPC/SBT: ampicillin/sulbactam; CEZ: cefazolin; BT: body temperature; CRP: C-reactive protein

She was diagnosed with pleurisy associated with MSSA pneumonia. A 20 Fr double-lumen chest tube was inserted into her right thoracic cavity and chest drainage was initiated on day 20. Because lytic agents were difficult to obtain, they were not administered, and only intrapleural lavage was performed. In addition, ampicillin/sulbactam was administered empirically on day 20. Her condition improved; therefore, the antimicrobial agent was changed to amoxicillin/clavulanic acid on day 34, and she was discharged with a plan of continuing antimicrobials for six to eight weeks according to the treatment of bacterial pleurisy. Figure 4 shows her clinical course from admission to discharge.

## Discussion

The efficacy of tocilizumab has been demonstrated in patients with severe COVID-19, and, in recent years, the use of tocilizumab, antivirals, and steroids has been recommended for the treatment of severe COVID-19 [5,6]. Both patients, treated with steroids and tocilizumab for severe COVID-19 pneumonia and considered immunosuppressed, developed severe infections with lung abscesses and pleurisy approximately 10 days after receiving tocilizumab.

The frequency of superinfection with common bacteria in COVID-19 pneumonia is reported to be approximately 3.5%-7.0% [1,2], and the complication rate of secondary infection with common bacteria is 14.3% [2]. Bacterial infections are particularly common in patients with severe COVID-19 admitted to the intensive care unit; superinfections occur in 44.6% [7], and secondary infections occur in 39.8% [8]. Corticosteroids, tocilizumab, and broad-spectrum antibiotics have been identified as risk factors for the development of superinfections in severe COVID-19 patients [7]. *P. aeruginosa* and *S. aureus* account for 25%-29% and 22%-24% of secondary infections caused by common bacteria, respectively, and most *S. aureus* cases are detected in blood cultures. Secondary infections with *P. aeruginosa* and *S. aureus* in influenza have been reported to occur in 35% and 17% of cases, respectively, with a significantly higher rate of concomitant *S. aureus* infections in COVID-19 [9]. Neutrophil extracellular traps (NETs) are important mediators of tissue damage in inflammatory diseases. SARS-CoV-2 directly induces the release of NETs by healthy neutrophils and promotes lung epithelial cell death [10]. We suspect that the damage to lung epithelial cells caused by SARS-CoV-2 may be the cause of bacterial infections, including those caused by *S. aureus*. Systemic corticosteroids and tocilizumab administration have been reported not to increase the risk of superinfection or secondary infections in patients with severe COVID-19 [8].

Tocilizumab is a monoclonal antibody targeting interleukin-6, which plays a central role in inflammatory responses such as fever by inducing the production of CRP, differentiation of B cells into antibody-producing cells, differentiation of cytotoxic T cells, leukocytosis, and platelet proliferation, thereby causing acute-phase responses and other inflammatory reactions [11]. Although the addition of tocilizumab to dexamethasone did not increase superinfection [12], those who received two doses of tocilizumab were reported to have a higher risk of superinfection than those who received one dose of tocilizumab [13]. A weakened immune system often underlies the severity of COVID-19, and tocilizumab use may increase the risk of secondary bacterial infections [14]. Tocilizumab may also mask inflammatory responses, such as fever and CRP production, due to secondary infections, potentially delaying the diagnosis and treatment of severe infections [11]. However, CRP levels are slightly elevated during severe bacterial infections [15]. Even if the fever and inflammatory reactions are slightly elevated, the possibility of serious bacterial infections (particularly *P. aeruginosa* and *S. aureus*) must be considered when treating severe COVID-19.

## Conclusions

We encountered a case of lung abscess and bacterial pleurisy secondary to severe COVID-19. Patients treated with tocilizumab for severe COVID-19 may be complicated by severe bacterial infections and the inflammatory response may be masked by tocilizumab. Even slight inflammation should be noted and treated with caution.
